# Is It Easy to Synchronize Our Minds When We Are Forced to Cooperate?

**DOI:** 10.3390/brainsci9100282

**Published:** 2019-10-18

**Authors:** Ángel Romero-Martínez, Alejandro Rodríguez, Luis Moya-Albiol

**Affiliations:** 1 Department of Psychobiology, University of Valencia, 46010 Valencia, Spain; 2 Biomedical Imaging Research Group (GIBI230), La Fe Health Research Institute, 46026 Valencia, Spain; alrodor.gibi230@gmail.com (A.R.); Luis.Moya@uv.es (L.M.-A.)

**Keywords:** competition, cooperation, gender, psychophysiology, synchronization

## Abstract

There is increasing scientific interest in elucidating the biological mechanisms underlying cooperative behaviors. Humans have developed a high degree of complexity in their cooperativity, which has been defined as hyper-cooperativity. An interesting biological marker to study how two individuals are emotionally linked when they cooperate is their psychophysiological synchronization (the overlapping of signals as indicators of Autonomous Nervous System activation). Hence, the main aim of this study was to explore participants’ psychophysiological synchronization, based on electrocardiograms (ECG) and galvanic skin response (GSR) signals in a sample of strangers who were set up to cooperate (*n* = 29 pairs of same sex strangers; mean age = 20.52 ± 1.72), compared to participants who were forced to compete (*n* = 22 pairs of same sex strangers; mean age = 20.45 ± 1.53) in a laboratory setting. Moreover, the roles of the participants’ gender and the outcomes (positive or negative) obtained in the cooperation were examined as potential moderators of this psychophysiological synchronization. Results showed a progressive increase in ECG and GSR signal synchronization in participants who cooperated, reaching the highest levels of synchronization during the recovery period. Moreover, cooperation induced higher GSR synchronization in comparison with competition. Finally, although gender played an important role in the psychophysiological synchronization during cooperation (women presented the highest overlapping of GSR signals), feedback about the participants’ performance was not significantly associated with their psychophysiological synchronization. Therefore, research in this field would help us to understand more about the body’s physiological responses to different types of social interactions, such as cooperation and competition, providing an opportunity to establish interaction strategies that would be physiologically desirable.

## 1. Introduction

Only a few species show cooperative strategies by achieving a shared goal between non-consanguineous members of the same species [1,2], which in turn increases the reproductive lifespan of these species [3]. Nevertheless, humans’ cooperative behaviors are different from those of other species and are to some extent idiosyncratic. In this regard, human cooperation tends to be sustained by complex cognitive processes, morality, and cumulative culture and technology, defined as hyper-cooperativity [4]. It has been suggested that this hyper-cooperativity characterized our *Homo sapiens* ancestors. In fact, they defeated Neanderthals in their competition for survival due to *Homo sapiens’* ability to build complex societies among their members. In addition, their survival might be explained by advanced communication and empathic abilities, which, in turn, increased the efficiency of *Homo sapiens’* work teams in yielding greater benefits [5]. Thus, it makes sense to think that these kinds of behaviors persist today and have reached unexpected levels of complexity.

As mentioned above, cooperation and the facilitation of coalition building require an advanced cognitive-emotional system (e.g., the ability to adopt another’s perspective, emotionally connect with others, experience concern…) in order to promote success in these social interactions [6,7,8,9,10]. Thus, the development of mature empathy might make individuals prone to cooperate [11]. Conversely, being forced to cooperate with strangers, especially for individuals with poor empathic abilities, could be extremely stressful and decrease the future likelihood of cooperating [7].

A strong attachment among group members (e.g., high affiliation, rapport, improvements in group dynamics…) leads to an increase in their psychophysiological synchrony. In other words, the psychophysiological time series of different individuals presents a significant overlap and/or coordinated dynamics when rapport within a group increases [12,13]. This could be a way to study how humans are emotionally linked and/or involved with each other (e.g., a couple) [14,15]. However, only two studies have analyzed the synchronization between individuals who cooperate by employing autonomic nervous system (ANS) markers such as heart rate (HR) and galvanic skin responses (GSR) [12,16]), which might offer information about their emotional regulation and/or its underlying processes [17,18,19,20].

One of these studies concluded that an increase in HR synchronization between two individuals who were forced to cooperate entailed higher levels of interpersonal trust between them [16]. In this experiment, participants were forced to cooperate in two laboratory conditions (half of the group in each condition), and their HR signals were continuously registered throughout the experiment. One of these conditions consisted of building different car models (based on the experimenter’s instructions) with LEGO pieces during four consecutive sessions (the control condition) in dyads of participants (they did not specify whether they were same sex dyads or not); meanwhile, the other experiment consisted of alternating the previously mentioned LEGO-car building sessions with public good game (PGG) sessions. In this case, HR synchrony was higher in the condition that combined LEGO-car building plus PGG than in the control condition. Moreover, the synchronization was higher during the PGG in relation to the participants’ expectations of their partner’s investment returns, but it was unrelated to their own investment.

Vanutelli et al. [16] demonstrated that GSR synchrony between the partners increased after receiving feedback on their performance, but the kind of feedback received (positive or negative) was not important [16]. In this study, same-sex dyads of participants were set to perform a common computer task (a simple task for sustained selective attention). In order to prevent any visual contact between participants, they were separated by a dark screen. To analyze the role of the performance feedback in synchronization, the experimenters constantly offered feedback about their cooperative performance, even though the feedback was pre-established; in other words, the result of the cooperation was manipulated, and the feedback provided was not real.

Although both experiments were interesting and offered valuable conclusions, they did not compare synchronization during cooperation and competition, which could be considered somewhat opposite behaviors. In fact, competition is a social behavior with which we try to obtain goals individually [21]. Furthermore, as previously demonstrated [8,9,10], it is necessary to study how participants’ gender and the outcome obtained affect their psychophysiological synchronization. Thus, the relationship between these variables and the previously mentioned autonomic markers still needs to be clarified.

Our main aim was to check for participants’ psychophysiological synchronization (HR and GSR) in a sample of strangers who were forced to cooperate in a laboratory context, compared to a same-sex dyad group forced to compete. Moreover, the role of the participants’ gender and the outcome (positive or negative) obtained in the cooperation were examined as potential moderators of this psychophysiological synchronization. Based on the Mitkidis et al. [12] and Vanutelli et al. [16] results, we hypothesized that participants who cooperated would have higher HR and GSR synchronization than those who competed. Second, regarding the role of gender, there was a lack of data in the literature on which to base a hypothesis. However, an important number of studies showed that women tend to be more cooperative than men [8,9,10,22], and so we hypothesized that women who cooperated would have higher psychophysiological synchronization than the other participants, especially men who compete. Finally, Vanutelli et al. [16] demonstrated that synchronization increases after receiving feedback on the performance (positive as well as negative), and so we would expect to find a significant relationship between the cooperative outcome and the psychophysiological synchronization.

## 2. Materials and Methods

### 2.1. Participants

The final sample included in our study consisted of 102 healthy young adults from the province of Valencia (Spain). In total, 56 men (mean age = 20.60 ± 1.73) and 46 women (mean age = 20.33 ± 1.24) were included. All of them were college students and right-handed. In the initial session, we collected a general self-report about habits and health. Afterwards, we carefully selected those participants who did not have drug use disorders or suffered from mental or other disorders (scoring below 5 on each scale on the General Health Questionnaire (GHQ-28)). Moreover, the potential effects of hormonal fluctuations on females’ psychological and mood variables were controlled by including only those females who presented a 3 month history of regular cycles and avoided the use of oral contraceptives [23].

Following the university ethics committee and Declaration of Helsinki ethical principles for human research, all of the participants included in our study voluntarily agreed to participate and signed an informed consent.

### 2.2. Procedure

Initially, anthropometric and demographic variables were collected from each participant. Moreover, their activities were registered during the two hours before their attendance at the session and the previous night.

After collecting the aforementioned information, participants were taken to a sound-proof, temperature-controlled (21 °C ± 2 °C) room where the experimental sessions took place. The sample was randomly allocated to two conditions, cooperation (16 pairs of men and 13 pairs of women) or competition (12 pairs of men and 10 pairs of women), to build a copy of a model house with LEGO pieces. During each session, two participants of the same gender (who did not know each other) were seated across from each other to perform one of the previously mentioned tasks (see for details [8,9,10,24,25]). During the experiment, which lasted approximately 30 min, electrocardiogram (ECG) and GSR parameters were continuously registered, but divided into four periods: resting, preparatory, task, and recovery (post-task), each lasting 10 min, with the exception of preparation which took 5 min. After the experiment ended, experimenters offered insight into the participants’ performance: win vs. lose (for the competitive task) and positive vs. negative (for the cooperative task and working alone). Moreover, they completed self-reports to assess empathic and cooperative abilities.

### 2.3. Electrophysiological Signals

ECG and GSR were registered by BIOPAC Systems Inc. (Santa Barbara, CA, USA) with data acquisition hardware (MP150) and data storage software (AcqKnowledge 4.2 for Windows, Biopac Systems, Montreal, QC, Canada).

HR and GSR determinism were studied by using MATLAB R2015b (The Mathworks Inc., Natick, MA, USA) and the Cross-Recurrence Plot Toolbox [26,27,28,29] with custom MATLAB scripts.

Prior to the synchronization analysis, each participant’s signals were segmented into the four periods (resting, preparatory, task, and recovery). After that, the HR and GSR datasets were down sampled to 4 Hz.

A recurrence quantification analysis (RQA) was applied in order to assess the HR and GSR synchrony between each pair of participants in each period. RQA allowed us to extract the determinism feature, which can test the relationship between time series and measure their degree of synchrony [12], thus assessing interpersonal cooperation. 

### 2.4. Self-Reports

The empathy quotient (EQ) consists of 60 items distributed on a Likert scale from 0 to 2. Forty items are related to empathy, whereas the remaining 20 control-items did not count in obtaining the total score. The higher the score, the greater the empathy [30].

Cooperativity was assessed with the cooperation subscale of the revised Spanish version [31], ‘temperament and character inventory’ [32]. It consists of 37 items rated on a Likert scale from 1 to 5, grouped in six subscales: social tolerance, empathy, altruism, compassion, integrity, and fellowship, and a final score was obtained from the total score of the above.

### 2.5. Statistical Analysis

The Shapiro–Wilk test was employed to examine whether the variables included in our study followed a normal distribution (*p* < 0.05). After checking for a normal distribution of the data, parametric tests were applied for the later analyses. Initially, t-tests and chi-square tests were employed to check for differences between the groups on socio-demographic variables and determinism parameters. 

Second, the Friedman test was performed to check for the effectiveness of the tasks in eliciting changes in synchronicity in the HR and GSR variables in the total sample and in each of the groups included in our study. Afterwards, the area under the curve with respect to the ground (AUC) was calculated for HR and GSR determinism. In this regard, we decomposed the total area under the curve as described by the participants’ HR and GSR determinism into simple triangles and rectangles. After that, we combined the area of each triangle and rectangle into a single formula [33,34,35,36]. For the calculation of differences between groups in area under a curve (AUC), we employed Mann–Whitney analyses, with ‘type of task’ (cooperation vs. competition) as between-subject factors.

Finally, Kendall rank correlation was employed to check for relationships between the AUC and gender (dummy coded as 0 = women and 1 = men) and the outcome obtained in the cooperation group (dummy coded as 0 = cooperation and 1 = competition). In order to avoid false positive errors, the Bonferroni adjustment for multiple comparisons was used. Specifically, we established the significance level at 0.013, given the number of comparisons made in our study.

We performed statistical analyses employing IBM SPSS (Version 24.0; IBM SPSS, Armonk, NY, USA), with results equal to or below *p* = 0.05 considered significant.

## 3. Results

There were no differences between the cooperation and competition groups in age (20.52 ± 1.72 and 20.45 ± 1.53, respectively), gender distribution ((women 44% and men 56%) and (women 41% and men 59%)), or menstrual cycle phase ((luteal 52%, follicular 38% and menstruation 10%) and (luteal 50%, follicular 25% and menstruation 25%)). Moreover, there were no differences in empathy (47.09 ± 9.08 and 44.64 ± 9.06, respectively) or cooperativity (136.90 ± 16.04 and 139.23 ± 14.50, respectively) between groups.

### 3.1. Effectiveness of the Laboratory Task in Eliciting ECG and GSR Synchronicity

**Figure 1:**
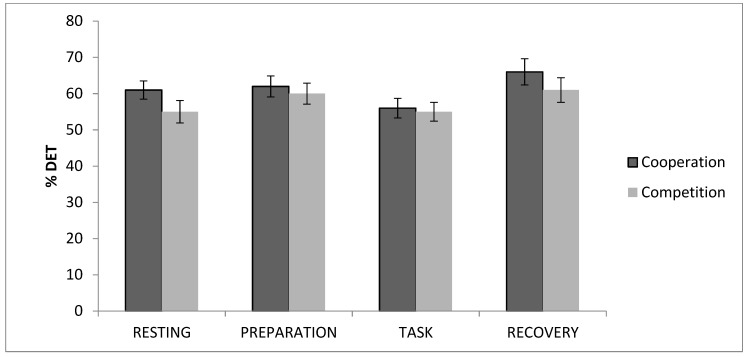
Electrocardiogram (ECG) percentage of determinism values for cooperation and competition groups.

Regarding HR determinism, there was a significant effect of ‘time’ on determinism, *χ*2(3) = 18.60; *p* = 0.000, in the total sample. After dividing the sample into groups, a significant effect of ‘time’ was only found in the cooperation group, *χ*2(3) = 17.87; *p* = 0.000. In the cooperation group, determinism values significantly increased from baseline to the preparation period. Afterwards, values decreased from this period to the task period, increasing from this point to the recovery period (*p* < 0.05, for all) (Figure 1). Finally, it should be noted that significant differences were found between the resting and posterior periods (*p* < 0.05, for all).

**Figure 2:**
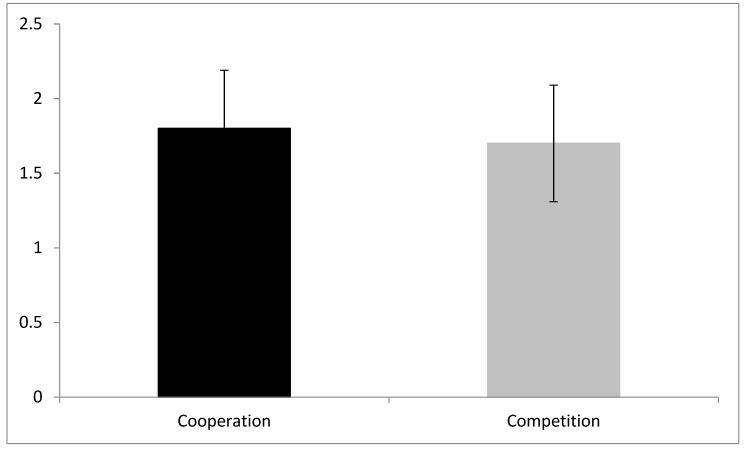
Heart rate (HR) area under the curve with respect to the ground (AUC) percentage of determinism values for cooperation and competition groups.

Regarding the HR determinism AUC values, there was no significant ‘group’ effect on determinism values (Z = −1.01; *p* = 0.313) (Figure 2).

**Figure 3:**
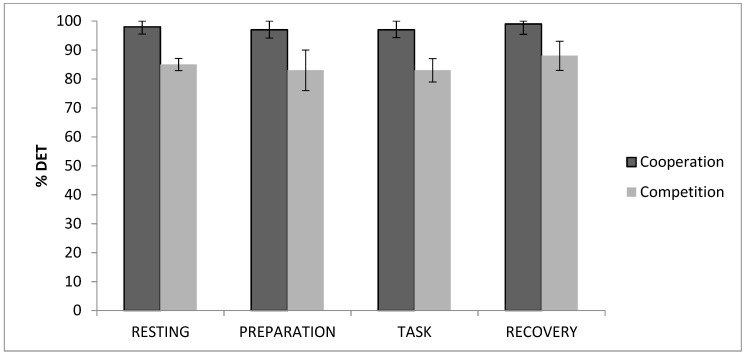
Galvanic skin response (GSR) percentage of determinism values for cooperation and competition groups.

With regard to GSR determinism, a significant effect of ‘time’ was found on determinism in the total sample, *χ*2(3) = 37.75; p = 0.000. After dividing the sample, a significant effect of ‘time’ was found in the cooperation group, *χ*2(3) = 23.19; *p* = 0.000, and the competition group, *χ*2(3) = 18.81; *p* = 0.000. For the cooperation group, determinism values significantly increased from the task to the recovery period (*p* < 0.05). However, participants who competed showed a significant decrease in determinism values from baseline to the task period, increasing from this point to the recovery period (*p* < 0.05, for all). Lastly, significant differences were also found between the resting and posterior periods (*p* < 0.05, for all) (Figure 3).

**Figure 4:**
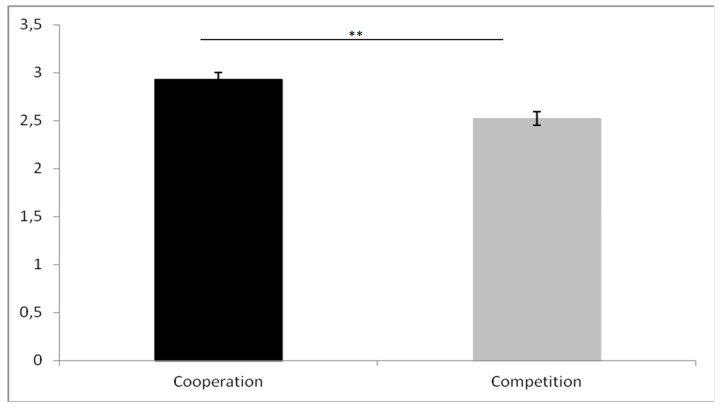
GSR AUC percentage of determinism values for cooperation and competition groups ** (*p* < 0.01).

Regarding GSR determinism AUC values, there was a significant ‘group’ effect on determinism AUC values (Z = −3.60; *p* < 0.001). The cooperation groups presented higher values than the competition groups (Figure 4).

### 3.2. Relationships between Determinism, Gender and Outcome

A significant and negative association was only found between GSR AUC determinism and gender (*τ* = −0.420, *p* = 0.007), with women being associated with higher GSR AUC determinism.

## 4. Discussion

Results showed a progressive increase in HR and GSR signal synchronization in participants who cooperated, reaching the highest levels during the recovery period. Moreover, the cooperation group presented higher GSR synchronization between the pairs of participants compared to individuals who competed. Finally, although gender demonstrated an important role in the psychophysiological synchronization in the cooperation condition (women presented the highest GSR overlapping), feedback about the participants’ performance was not significantly associated with psychophysiological synchronization in the cooperation group.

Our first hypothesis was that those participants who cooperated with a positive outcome would have higher HR and GSR synchronization than those who competed [12,16]. Nevertheless, in general, the findings did not completely support this hypothesis. In fact, differences were only found between the groups on GSR, with participants who cooperated presenting higher total GSR synchronization than those who competed. Moreover, it should be noted that in both cases (ECG and GSR), participants presented an increase in their synchronization from resting to the preparation period when researchers explained that they would cooperate (i.e., during the preparation period). However, synchronization decreased during the task period in participants who cooperated. This result may be because this is a critical period for participants in satisfactorily managing stress, which involves intense sympathetic nerve activation (SNA). Conversely, the participants who competed experienced a decrease in their GSR synchronization from resting to the preparation period. The intense SNA activation implies that the heart rate variability (HRV) of each participant reduced the probability of harmonizing their psychobiological signals. In fact, this finding is supported by the fact that the maximum level of synchronization appeared during the recovery period. In this regard, it is highly likely that competition implies different feelings and HRV functioning for winners and losers [8,9,10], which may produce low congruency in ECG signals. Thus, it seems logical to imagine that cooperation (unless compared to competition) produces stronger links and closer interpersonal relationships between individuals [37].

Regarding the second hypothesis, we suggested that women who cooperated would have higher psychophysiological synchronization than the other participants, especially men who competed. Our data partially support this idea. In fact, women reported higher GSR synchronization, but no significant effect was found for the ECG signal. As previously suggested, gender seems to play an important role in cooperation [8,9,10,24,25]. Thus, it makes sense to think that women present a natural tendency to easily establish emotional links with other individuals, which might be facilitated by their superior empathic and cooperation skills [22].

Finally, Vanutelli et al. [16] suggested that offering feedback (regardless of its positive or negative value) results in an increase in synchronization levels, and our results showed that the outcome’s value did not play a relevant role in psychophysiological synchronization. Nevertheless, there are important differences between the studies, especially regarding methodological questions (reduced sample sizes, different statistical strategies to measure synchronization, different laboratory tasks, etc.). However, there is no doubt that previous research in this field alongside our laboratory task indicated that the value of the outcome (winner vs. loser or positive vs. negative) leads to differences in biological markers [8,9,10,24,25]. Thus, this variable should be considered in future research in order to determine whether the value of the feedback is important or not.

To the best of our knowledge, this is the first study to examine whether different types of social strategies produce psychophysiological synchronization between individuals. Despite the study’s strengths, some limitations should be considered when interpreting the results. First, the cross-sectional and correlational nature of the study makes it difficult to establish causality in the results. Moreover, our data were obtained from healthy young adults, and we only analyzed two types of social interaction. Another limitation of the current study is that not all of the individuals went through both conditions. In fact, we compared different individuals, and so it is difficult to conclude whether individuals’ synchronization varied across different social interactions. Future research should consider the association between ANS and electroencephalographical signals, in order to understand the meaning of this synchronization through the interpretation of brain activation. Furthermore, it would be advisable to study the role of specific hormones such as oxytocin, a hormone related to empathy [38] and probably also to cooperation [39], testosterone, and/or cortisol in psychophysiological synchronization. This information will help to create a broader understanding of cooperation and develop a holistic model, as previous research has done in the case of competition.

## 5. Conclusions

To conclude, because our results were obtained in a laboratory context with a non-athletic population, they could be generalizable to many individuals, compared to studies based only on an athletic population. Furthermore, research in this field would help us to understand more about the body’s physiological responses to different types of social interactions, such as cooperation and competition, providing an opportunity to establish interaction strategies that would be physiologically desirable. Furthermore, these results might be useful as a guide to developing effective biofeedback interventions to promote attachment between co-workers and couples.

## References

[1] Clutton-Brock T. (2009). Cooperation between Non-Kin in animal societies. Nature.

[2] Schaik C.P., Kappeler P.M., Kappeler P.M., Schaik C.P. (2006). Cooperation in primates and humans: Closing the gap. Cooperation in Primates and Humans: Mechanisms and Evolution.

[3] Bergmüller R., Taborsky M. (2007). Adaptive behavioural syndromes due to strategic niche specialization. BMC Ecol..

[4] Burkart J.M., Allon O., Amici F., Fichtel C., Finkenwirth C., Heschl A., Huber J., Isler K., Kosonen Z.K., Martins E. (2014). The evolutionary origin of human Hyper-Cooperation. Nat. Commun..

[5] Mellars P., French J.C. (2011). Tenfold population increase in western europe at the Neandertal–to–Modern human transition. Science.

[6] Gilin D., Maddux W.W., Carpenter J., Galinsky A.D. (2013). When to use your head and when to use your heart: The differential value of perspective-taking versus empathy in competitive interactions. Personal. Soc. Psychol. Bull..

[7] Romero-Martínez Á., Sariñana-González P., Moya-Albiol L. (2017). Is Low Empathy a Reason to Refuse to Cooperate with Strangers?. Am. J. Psychol. Behav. Sci..

[8] Sariñana-González P., Romero-Martínez Á., Moya-Albiol L. (2017). Does being a Stranger make it Difficult to Cooperate?. Span. J. Psychol..

[9] Sariñana-González P., Romero-Martínez Á., Moya-Albiol L. (2018). Cooperation Between Strangers in Face-to-Face Dyads Produces More Cardiovascular Activation Than Competition or Working Alone. J. Psychophysiol..

[10] Sariñana-González P., Romero-Martínez Á., Moya-Albiol L. (2016). Cooperation Induces an Increase in Emotional Response, as Measured by Electrodermal Activity and Mood. Curr. Psychol..

[11] Rumble A.C., Van Lange P.A.M., Parks C.D. (2010). The benefits of empathy: When empathy may sustain cooperation in social dilemmas. Eur. J. Soc. Psychol..

[12] Mitkidis P., McGraw J.J., Roepstorff A., Wallot S. (2015). Building trust: Heart rate synchrony and arousal during joint action increased by public goods game. Physiol. Behav..

[13] Wiltermuth S.S., Heath C. (2009). Synchrony and cooperation. Psychol. Sci..

[14] Chaspari T., Baucom B., Timmons A.C., Tsiartas A., Del Piero L.B., Baucom K.J., Georgiou P., Margolin G., Narayanan S.S. Quantifying EDA synchrony through joint sparse representation: A case-study of couples’ interactions. Proceedings of the 2015 IEEE International Conference on Acoustics, Speech and Signal Processing (ICASSP).

[15] Coutinho J., Oliveira-Silva P., Fernandes E., Gonçalves O.F., Correia D., Perrone Mc-Govern K., Tschacher W. (2018). Psychophysiological Synchrony During Verbal Interaction in Romantic Relationships. Family Process..

[16] Vanutelli M.E., Gatti L., Angioletti L., Balconi M. (2017). Affective Synchrony and Autonomic Coupling during Cooperation: A Hyperscanning Study. BioMed Res. Int..

[17] Critchley H.D., Eccles J., Garfinkel S.N. (2013). Interaction between cognition, emotion, and the autonomic nervous system. Handbook of Clinical Neurology.

[18] Kreibig S.D. (2010). Autonomic nervous system activity in emotion: A review. Biol. Psychol..

[19] Mauss I.B., Robinson M.D. (2009). Measures of emotion: A review. Cogn. Emot..

[20] Feldman R., Magori-Cohen R., Galili G., Singer M., Louzoun Y. (2011). Mother and infant coordinate heart rhythms through episodes of interaction synchrony. Infant Behav. Dev..

[21] Mazur A. (1985). A biosocial model of status in Face-to-Face primate groups. Soc. Forces.

[22] Balliet D., Li N.P., Macfarlan S.J., Van Vugt M. (2011). Sex differences in cooperation: A Meta-Analytic review of social dilemmas. Psychol. Bull..

[23] Gómez-Amor J., Martínez-Selva J., Román F., Zamora S., Sastre J. (1990). Electrodermal activity, hormonal levels and subjective experience during the menstrual cycle. Biol. Psychol..

[24] de Andrés-García S., González-Bono E., Sariñana-González P., Sanchis-Calatayud M.V., Romero-Martínez Á., Moya Albiol L. (2011). La valoración del resultado modula la respuesta del cortisol a una tarea cooperativa de laboratorio en mujeres. Psicothema.

[25] Moya-Albiol L., De Andrés-García S., Sanchis-Calatayud M.V., Sariñana-González P., Ruiz-Robledillo N., Romero-Martínez A., González-Bono E. (2013). Psychophysiological responses to cooperation: The role of outcome and gender. Int. J. Psychol..

[26] Boccaletti S., Latora V., Moreno Y., Chavez M., Hwang D.-U. (2006). Complex networks: Structures and dynamics. Phys. Rep..

[27] Little M., McSharry P., Roberts S., Costello D., Moroz I. (2007). Exploiting Nonlinear Recurrence and Fractal Scaling Properties for Voice Disorder Detection. Biomed. Eng. Online.

[28] Marwan N., Romano M.C., Thiel M., Kurths J. (2007). Recurrence Plots for the Analysis of Complex Systems. Phys. Rep..

[29] Marwan N., Donges J.F., Zou Y., Donner R.V., Kurths J. (2009). Complex network approach for recurrence analysis of time series. Phys. Lett. A.

[30] Baron-Cohen S., Wheelwright S. (2004). The empathy quotient (EQ). An investigation of adults with asperger syndrome or high functioning au ism, and normal sex differences. J. Autism Dev. Disord..

[31] Gutiérrez-Zotes J.A., Bayón C., Montserrat C., Valero J., Labad A., Clo-ninger C.R., Fernández-Aranda F. (2004). Inventario del Tempera-Mento y el Carácter-Revisado (TCI-R). Baremación y datos normativos en una muestra de población general. Actas Españolas Psiquiatr..

[32] Cloninger C.R., Svrakic D.M., Przybeck T.R. (1993). A psychobiological model of temperament and character. Arch. Gen. Psychiatry.

[33] Fekedulegn D.B., Andrew M.E., Burchfiel C.M., Violanti J.M., Hartley T.A., Charles L.E., Miller D.B. (2007). Area under the curve and other summary indicators of repeated waking cortisol measurements. Psychosom. Med..

[34] Grice J.E., Jackson R.V. (2003). Letter to the editor: Two formulas for computation of the area under the curve represent measures of total hormone concentration versus Time-Dependent change. Psychoneuroendocrinology.

[35] Romero-Martínez A., Lila M., Williams R.K., González-Bono E., Moya-Albiol L. (2013). Skin conductance rises in preparation and recovery to psychosocial stress and its relationship with impulsivity and testosterone in intimate partner violence perpetrators. Int. J. Psychophysiol..

[36] Romero-Martínez A., Lila M., Conchell R., González-Bono E., Moya-Albiol L. (2014). Immunoglobulin A response to acute stress in intimate partner violence perpetrators: The role of anger expression-out and testosterone. Biol. Psychol..

[37] Jackson J.C., Jong J., Bilkey D., Whitehouse H., Zollmann S., McNaughton C., Halberstadt J. (2018). Synchrony and Physiological Arousal Increase Cohesion and Cooperation in Large Naturalistic Groups. Sci. Rep..

[38] Takahashi T., Ikeda K., Ishikawa M., Kitamura N., Tsukasaki T., Nakama D., Kameda T. (2005). Interpersonal trust and social Stress-Induced cortisol elevation. Neuroreport.

[39] De Dreu C.K. (2012). Oxytocin modulates cooperation within and competition between groups: An integrative review and research agenda. Horm. Behav..

